# Factors Associated with the Refusal of Direct-Acting Antiviral Agents for the Treatment of Hepatitis C in Taiwan

**DOI:** 10.3390/medicina58040521

**Published:** 2022-04-07

**Authors:** Li-Jen Chang, Han-Cheng Chang, Po-Yueh Chen, Chi-Yi Chen, Kun-Feng Tsai, Koh-Kok Yean, Hsin-Yi Yang, Tsung-Hsien Chen, Pao-Ta Yu, Chu-Kuang Chou, Sheng-Hsuan Chen

**Affiliations:** 1 Division of Gastroenterology and Hepatology, Department of Internal Medicine, Ditmanson Medical Foundation Chia-Yi Christian Hospital, Chiayi 60002, Taiwan; cych07235@gmail.com (L.-J.C.); hdilwy7@gmail.com (P.-Y.C.); 5137ccy@gmail.com (C.-Y.C.); 04236@cych.org.tw (K.-K.Y.); 2 Department of Internal Medicine, Ditmanson Medical Foundation Chia-Yi Christian Hospital, Chiayi 60002, Taiwan; cych13794@gmail.com; 3 Obesity Center, Ditmanson Medical Foundation Chia-Yi Christian Hospital, Chiayi 60002, Taiwan; 4 Min-Hwei Junior College of Health Care Management, Tainan 73658, Taiwan; 5 Information Technology Department, Ditmanson Medical Foundation Chia-Yi Christian Hospital, Chiayi 60002, Taiwan; 12452@cych.org.tw; 6 Department of Computer Science & Information Engineering, National Chung Cheng University, Chiayi 62130, Taiwan; csipty@cs.ccu.edu.tw; 7 Clinical Trial Center, Ditmanson Medical Foundation Chia-Yi Christian Hospital, Chiayi 60002, Taiwan; 8 Gastroenterology and Hepatology Section, Department of Internal Medicine, An Nan Hospital, China Medical University, Tainan 709, Taiwan; tsai.kf@gmail.com; 9 Department of Medical Sciences Industry, Chang Jung Christian University, Tainan 711301, Taiwan; 10 Clinical Medicine Research Center, Ditmanson Medical Foundation Chia-Yi Christian Hospital, Chiayi 60002, Taiwan; cych13018@gmail.com

**Keywords:** chronic hepatitis C, direct-acting antiviral therapy, Taiwan’s National Health Insurance

## Abstract

*Background and Objectives*: Direct-acting antiviral agents (DAA) are a safe and highly effective treatment for hepatitis C virus (HCV) infection. However, the uptake of DAA treatment remains a challenge. This study aims to examine the reasons for DAA refusal among HCV patients covered by the Taiwan National Health Insurance system. *Materials and Methods*: This retrospective observational study covered the period from January 2009 to December 2019 and was conducted at a single hepatitis treatment center in Taiwan. This study involved chart reviews and phone-based surveys to confirm treatment status and refusal causes. To confirm treatment status, subjects with HCV without treatment records were phone-contacted to confirm treatment status. Patients who did not receive treatment were invited back for treatment. If the patient refused, the reason for refusal was discussed. *Results*: A total of 3566 patients were confirmed with DAA treatment; 418 patients (179 patients who were lost to contact or refused the survey and 239 patients who completed the survey of DAA refusal) were included in the no-DAA-therapy group. Factors associated with receiving DAAs were hemoglobin levels, hepatitis B virus co-infection, and regular gastroenterology visits. Meanwhile, male sex, platelet levels, and primary care physician visits were associated with DAA refusal. The leading causes of treatment refusal were multiple comorbidities, low health literacy, restricted access to hospitals, nursing home residence, and old age. The rate of DAA refusal remains high (10%). *Conclusions*: The reasons for treatment refusal are multifactorial, and addressing them requires complex interventions.

## 1. Introduction

Hepatitis C virus (HCV) is a leading cause of acute and chronic hepatitis, cirrhosis, and hepatocellular carcinoma, affecting approximately 130–150 million people worldwide [1]. The global prevalence of HCV infection in 2015 was in the range of 0.5% to 2.3% [2]. However, most infected patients progress to develop chronic HCV infection [3]. When HCV infection becomes chronic, treatment with antiviral therapy may reduce the risk of morbidity and mortality. New pan-genotypic direct-acting antiviral (DAA) therapy has been reported to cure >95% of HCV infection cases and to be associated with a low rate of adverse events [4,5,6] while achieving a sustained virological response [7,8,9]. DAAs have also been shown as highly effective at treating Taiwanese HCV patients and hepatitis B virus (HBV) co-infected patients [10]; many patients previously excluded from antiviral therapy are now eligible to receive DAAs.

DAA therapy is short-term and straightforward to administer and has improved safety and efficacy profiles compared to those of interferon-containing regimens that may have severe side effects [1]. In 2014, DAA therapy was approved by the European Medicines Agency, and interferon-free treatment was available for the first time. However, population-level effectiveness depends on the number of HCV patients receiving DAA therapy. Although up to 75% of HCV-infected patients are treated at specialized clinics, most patients defer therapy [11,12]. It is reported by an American study that 75% of 324 patients seen during 2001–2005 chose to defer therapy based on psychiatric issues or substance abuse at the time of their initial evaluation; approximately half of the patients returned for follow-up assessments, but only 13% eventually received treatment and had a trend toward lower SVR rates [13]. In a retrospective study by the United States Department of Veterans’ Affairs, 56.2% of 905 patients had undergone a specialist visit and 28% received DAAs [14]. Alcohol and drug use are among the most common reasons for DAA therapy refusal [14]. The asymptomatic nature of the infection, cultural mistrust, and other psychosocial, medical, and provider- and insurance-related factors may make HCV patients in the USA hesitant to undergo DAA therapy [15,16]. To find possible factors, most studies were conducted with chart reviews or insurance system reviews [16,17,18,19,20]. Patients might receive DAA treatment from different insurance programs or hospitals or through self-buy generic medications, and the determination of DAA treatment only by reviewing records may not represent real treatment status. We combined chart reviews and then phone confirmation for DAA treatment status.

The prevalence of HCV in Taiwan is 3.28% (1.8–5.5%) in the general population and >10% in several HCV hyperendemic areas, which is the highest in northeast Asia [21,22]. In 1995, Taiwan established a universal National Health Insurance (NHI) program. As of 2013, over 99.9% of Taiwan’s 23.4 million residents had been enrolled in this program [23]. All residents have an NHI card that allows health providers to access patients’ medical information, including details of visits, prescriptions, and vaccinations [24]. The implementation of a universal healthcare program has reduced health disparities among the citizens. Although DAA therapy is costly, oral drug treatments for HCV are covered by the NHI. This study aims to examine follow-up data from a regional hospital in Taiwan to identify barriers to treatment and reasons for HCV treatment refusal.

## 2. Materials and Methods

This retrospective observational study was conducted at the Chia-Yi Christian Hospital, the dominant HCV treatment center in southern Taiwan.

Data on patients with positive anti-HCV antibody or HCV RNA findings were extracted from electronic medical records from January 2009 to December 2019 [9]. We used phone contact for subjects without treatment records after the chart review in 2020. We confirmed their treatment status by phone, reminded HCV patients without further treatment, performed phone surveys for those who refused further treatment in 2020, and recorded all the results of the survey. Moreover, we retrospectively reviewed the clinical data from January 2009 to December 2019 to find possible clinical predictors. We did the analysis in 2021. The patients were divided into the following groups:

1. No-DAA treatment group: Patients with positive RNA findings without a record of DAA treatment or those who had positive anti-HCV antibody results but without further HCV RNA findings were contacted. Patients that confirmed they were not receiving treatment and those who refused further tests and treatments were included in this group. Patients who could not be contacted or refused the telephone survey were also included in this group, as in the previous chart review study [14].
2. DAA treatment group: The chart reviews of patients with a record of DAA treatment in our hospital and patients confirmed with DAA treatment out of our hospital during a phone interview were included in this group. All cases in the DAA group are solely from records review.

Patients were excluded from this study if they met any of the following criteria: spontaneous or treatment-related infection remission from interferon (negative HCV RNA findings after initial positive findings with or without DAA use), death, and insufficient data. Patients were eligible for this study if they were aged ≥18 years. For the no-DAA treatment group, we used the most recent laboratory data available in our hospital. For the DAA treatment group, we used the data before DAA treatment. This study was approved by the Institutional Review Board of Chiayi Christian Hospital (approval no. CYCH-IRB-2018027).

### 2.1. Data Collection and Analysis of Clinical Data

We collected the data that are commonly available in clinics to determine possible reasons for refusal. The data included (1) demographic characteristics: age, sex, HBV co-infection, primary care physician visits in 2019, gastroenterology visits in 2019, long-term drug use during 2019, and distance from the hospital (average transfer time to hospital); (2) data on clinical laboratory findings: hemoglobin levels (g/dL), platelet count (10^3^/μL), mean white blood cell count (10^3^/μL), prothrombin time (s), and the levels of aspartate aminotransferase (U/L), alanine aminotransferase (U/L), and creatinine (mg/dL); (3) Fibrosis-4 Index, calculated to estimate the risk of advanced fibrosis in the present sample [25]; (4) cirrhosis status based on imaging findings consistent with the characteristics of cirrhosis. Univariate and multivariate analyses were conducted.

### 2.2. DAA Refusal Phone-Based Survey to Confirm the Refusal Causes

Patients in 2009–2019 who did not have evidence of receiving DAA in their medical records to confirm whether or not they received DAA were queried for reasons for not receiving DAA. We contacted all patients with positive RNA findings without a record of DAA treatment and those with positive anti-HCV antibody findings without further HCV RNA data. The purpose of our records was to identify explanations for the lack of follow-up that could not be obtained from data. We confirmed the patients’ treatment history. If they had not received treatment, a study nurse invited them back to the hospital to complete the treatment. If they refused further treatment or RNA tests to confirm their treatment needs, the reason for this refusal was asked and recorded. Forgoing treatment for self-confessed health was classified as lower health literacy.

### 2.3. Statistical Analysis

All analyses were performed using SPSS version 17.0 (SPSS Inc., Chicago, IL, USA). Continuous variables were expressed as mean ± standard deviation, and categorical variables were expressed as count (percentage). Comparisons of continuous data between groups were tested by *t*-test. Differences in categorical data between groups were examined by Pearson’s chi-square test. Univariate and multivariate logistic regression models were used to identify factors associated with receptivity to DAA treatment among HCV patients. Multivariable logistic regressions for potential factors were derived from the electronic medical records. The results of the phone survey were presented as a proportion; *p*-values of <0.05 were considered statistically significant.

## 3. Results

**Figure 1:**
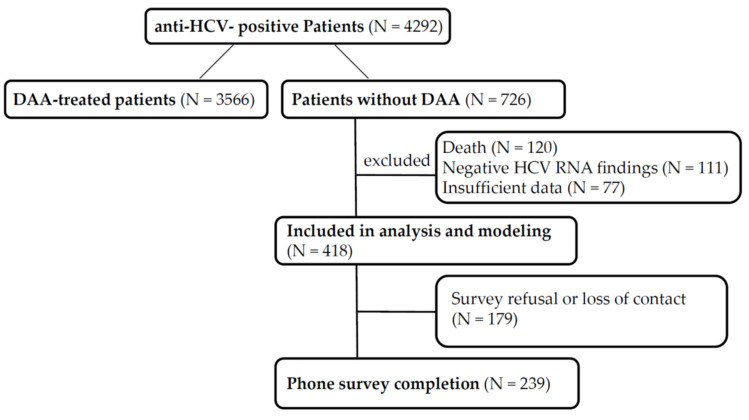
Flow diagram. DAA-treated patients were confirmed by records review, whereas patients without DAA were based on either phone confirmation of no desire to receive DAA or inability to reach the patient by phone.

A total of 4292 patients with positive anti-HCV antibody findings were identified; among them, 3566 patients had received DAA treatment at the gastroenterology and hepatology clinics in Chia-Yi Christian Hospital or another hospital, and their treatment status was confirmed either through medical records or the phone survey (Figure 1). A total of 308 patients were excluded due to death (N = 120), negative HCV RNA findings without DAA treatment (N = 111), or insufficient data (N = 77). The remaining 418 patients were confirmed as having positive anti-HCV antibody findings without undergoing further RNA testing for DAA treatment or as having positive anti-HCV antibody and positive RNA test findings without DAA treatment. These patients were included in the no-treatment group. The survey refusal rate or loss of contact rate was 42.8% (N = 179/418).

The patients’ characteristics are presented in Table 1. Patients that received DAA treatment had higher levels of hemoglobin (13.2 ± 2 g/dL vs. 12.7 ± 2.3 g/dL, *p* < 0.001), lower platelet (187.7 ± 70.3 10^3^/μL vs. 209.4 ± 86.4 10^3^/μL, *p* < 0.001) and mean white blood cell (6.7 ± 2.6 × 10^3^/μL vs. 7.2 ± 3.1 × 10^3^/μL, *p* = 0.001) counts, and higher rates of HBV co-infection (33.6% vs. 18.2%, *p* < 0.001) and cirrhosis (16.9% vs. 8.1%, *p* < 0.001) than their counterparts.

Among the patients who did not receive DAA treatment, 96.9% remained under clinical surveillance in 2019. The patients that received DAA treatment recorded more primary care physician visits in 2019 (18.9 ± 30.7 vs. 13.9 ± 26.4, *p* = 0.001) than their counterparts. Only 22.7% of untreated patients continued to visit gastroenterology clinics in 2019. Patients that received DAAs underwent more gastroenterology visits in 2019 (5.1 ± 4.3 vs. 0.8 ± 3.1, *p* < 0.001) than their counterparts.

### 3.1. Factors Associated with HCV Treatment

Statistically significant predictors of receiving DAA treatment were hemoglobin levels (*p* < 0.001), HBV co-infection (*p* < 0.001), cirrhosis (*p* < 0.001), proximity to hospital (<30 min) (*p* = 0.013), and gastroenterology visits in 2019 (*p* < 0.001). Meanwhile, platelet count (*p* < 0.001), mean white blood cell count (*p* < 0.001), and primary care physician visits in 2019 (*p*< 0.001) were associated with DAA treatment refusal (Table 2)**.**

In our multivariate model, we included covariates that were significant in univariate analyses as well as others that we had determined should be included a priori. In stepwise multivariate regression, high hemoglobin levels (adjusted odds ratio (OR) 1.20, 95% confidence interval (CI) 1.12–1.29, *p* < 0.001), co-infection with HBV (adjusted OR 1.85, 95% CI 1.35–2.54, *p* < 0.001), and gastroenterology visits in 2019 (adjusted OR 4.56, 95% CI 2.82–7.37, *p* < 0.001) were independent predictors of receiving DAA treatment (Table 2). In addition, the analysis revealed that male sex (adjusted OR 0.64, 95% CI 0.48–0.85, *p* = 0.002), platelet count (adjusted OR 0.998, 95% CI 0.996–1.00, *p* = 0.014), and primary care physician visits in 2019 (adjusted OR 0.07, 95% CI 0.04–0.14, *p* < 0.001) were associated with no-DAA treatment.

### 3.2. Common Reasons for DAA Treatment Refusal

**Figure 2:**
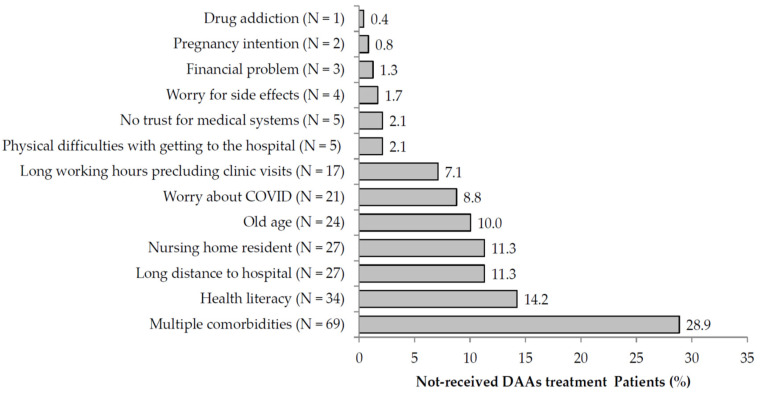
Implicit review findings: reasons for the lack of clinical follow-up on hepatitis C infection.

According to our phone survey, for patients who did not receive DAA treatment, a total of 239 patients provided their reasons for treatment refusal, including multiple comorbidities (*n* = 69, 28.9%), low health literacy (N = 34, 14.2%), distance from hospital (N = 27, 11.3%), residing at a nursing home (N = 27, 11.3%), old age (N = 24, 10.0%), concerns over the novel coronavirus disease 2019 pandemic (N = 21, 8.8%), long working hours precluding clinic visits (N = 17, 7.1%), physical difficulties with getting to the hospital (N = 5, 2.1%), lack of trust in medical systems (N = 5, 2.1%), concerns over adverse effects (N = 4, 1.7%), financial concerns (N = 3, 1.3%), intention to get pregnant (N = 2, 0.8%), and drug addiction (N = 1, 0.4%) (Figure 2). In the no-treatment group, some patients were lost to contact or refused to answer the survey (N = 179).

## 4. Discussion

In this retrospective cohort study, we have examined factors associated with receiving and refusing DAA treatment among HCV patients from a tertiary referral center under the Taiwan National Health Insurance system. In the present study, factors associated with receiving DAA treatment were hemoglobin levels, co-infection with HBV, and gastroenterology visits in 2019. However, male sex, platelet levels, and primary care physician visits in 2019 were the main factors associated with refusing DAA treatment. The reasons for refusing DAA treatment included insufficient health literacy, chronic illness, distance from the hospital, perceived time constraints, willingness to receive treatment, and concern over medical expenses based on our phone survey.

In most countries, patients might receive DAA treatment from different insurance programs, in hospitals, with self-buy generic medications without any insurance records, or even in different countries. A new self-report tool (HCV-AD) was recently developed to measure factors of intentional or unintentional adherence during HCV treatment to avoid drug resistance and treatment failure [26]. The chart or program-based reviews may not determine DAA treatment or not correctly. Our study combined chart reviews and phone confirmations to confirm DAA treatment status [16,17,18,19,20]. No other method can fully confirm the treatment status better than this combination of record review and personal contact. It is hard to determine the treatment status of those patients who cannot be contacted. Like the previous study, the subjects with treatment records and loss of contact were included in the no-treatment groups. Most chart or program review studies classified patients without treatment records into the no-treatment group [16,17,18,19,20]. We made phone confirmations for all the patients without treatment records; hence, the accuracy of treatment status was better in our study. The real treatment proportion might be a little higher because the lost-to-contact patients might have received or be receiving treatment.

Current HCV guidelines recommend treatment for all patients with chronic infection, except those with short life expectancies that cannot be remediated by treating HCV [27]. Herein, 89.5% of HCV-infected patients received DAA treatment under the Taiwan NHI system at a regional teaching hospital. The linkages to care of all HCV patients in Taiwan are generally good because of the high accessibility of specialists, affordable medical costs, and national HCV eradication programs, which have increased the proportion of DAA treatment. During the COVID-19 pandemic lockdown, telemedicine has emerged as a reliable tool and a novel alternative for the routine monitoring of patients receiving direct-acting antiviral drugs [28]. The linkage to care in patients who refuse to DAAs in our hospital included phone call-backs and outreach programs for specific groups. Before this analysis, we had conducted several outreach HCV treatment programs for prisoners, several nursing homes, and areas far from any HCV treatment centers.

The present refusal rate is 10.5%, only marginally higher than the rates of 2.5–15% reported in previous studies [29,30,31,32,33,34,35]. In a retrospective cohort study by the United States Department of Veterans, common reasons for the loss of follow-up were relocation (37.4%) and missed/canceled appointments (15.9%) [14]. The asymptomatic nature of infection and potential mistrust makes patients hesitant to undergo DAA in the Somalis [15]. In Australia, Canada, and other countries, the issues of persons who inject drugs (PWIDs) were faced [36,37]. Gender, human immunodeficiency virus (HIV), and ethnicity might cause the different rates of treatment acceptance [38]. During our phone survey, the common reasons for treatment refusal or follow-up were underlying causes and health literacy. The data showed the difference between different countries and the importance of domestic data.

Receiving DAA treatment was associated with sociodemographic and clinical characteristics among patients in Taiwan. Our phone survey revealed that approximately half of the eligible patients refused DAAs due to chronic illness, nursing home residency, and old age. The chronic illnesses contributing to the DAA treatment refusal were very complex. Most patients had multiple chronic illnesses such as dementia, chronic kidney diseases, heart failure, or cancers. In addition, 14% of the participants refused treatment due to low health literacy. In an urban core community center in the United States, 69% of patients face obstacles in starting DAAs therapy, including: psychosocial, medical, and provider- and insurance-related obstacles, among others. Specifically, failure to keep appointments (33%), abuse of active substances (8%), and failure to obtain laboratory tests (5%) are common barriers to treatment [16]. In the US Veterans Administration health care system, other reasons for treatment resistance included waiting for newer therapy (38.8%), comorbidities (25.9%), and alcohol/drug abuse (24.7%) [14]. The uptake of DAA therapy in America was low in 2014, especially so among minority and Medicaid patients [39]. Among patients in general US health care settings, higher annual income, higher Fibrosis-4 score, genotype 2 infection, pre-2014 treatment failure, and HIV co-infection were associated with DAA initiation [39].

The present results show that hemoglobin levels, HBV co-infection, and the number of gastroenterology visits in 2019 are independently associated with receiving DAAs; other factors associated with this outcome are health literacy, chronic illness, proximity to hospitals, and perceived time restrictions. A large proportion of DAA refusal patients are elderly with multiple comorbidities or were nursing home residents after DAA treatment started to be covered by national insurance. DAA treatment is generally not recommended in patients with limited life expectancy due to non-liver-related comorbidities, e.g., at the end of life. Considering life expectancy, treating HCV with DAA may not be beneficial for this group of patients. Gaps in health literacy may contribute to the lack of follow-up in HCV tests and DAA treatment. Interventions such as provider education on HCV screening and treatment may help overcome these barriers. The patients with HCV and HBC co-infection would have worse liver outcomes [40]. Most co-infection patients were much more eager to receive DAA treatment. In Taiwan, patients have financial concerns despite HCV treatment being covered by the NHI, and proximity to hospitals remains a problem. Lower Hb level correlated with DAA refusal. The reason for Hb levels is not clear. We postulate that patients who have more chronic illnesses or older age may have anemia, and they may refuse to receive treatment due to these diseases. The refusal rate in our cohort was low, and we believe a significant proportion of the remaining untreated patients had no treatment because of some medical reasons. This point is supported by our phone survey. It is well known that low platelet levels correlate with the presence of cirrhosis, and we found that patients who had HCV-related cirrhosis were more willing to treat HCV. The reasons for treatment refusal vary; they tend to be multifactorial and are unlikely to be overcome by a single intervention. Most patients (96.9%) who did not receive DAA continued to attend hospital and clinic visits in 2019, suggesting that patients are likely to receive care even if they refuse DAA treatment. However, challenges associated with low health literacy may be substantial.

This study has several limitations. Our medical record review was limited to a single center; consequently, the present findings may not generalize to patients at other hospitals. In addition, despite our attempts to confirm HCV treatment history, a total of 179 participants could not be reached by phone or refused to participate in the phone survey. Some of these patients may have received treatment at another hospital or acquired generic medications through other means. We did not perform a comprehensive questionnaire-based assessment of the reasons for treatment refusal; the phone-based survey may have failed to identify the real reason for DAA refusal. We may not explore the reason detailed for each subject, such as the comorbidities involved in the refusal of DAA, the level of education of patients who refused DAA, and worry as to side effects. Identifying the exact reasons for treatment refusal remains a challenge. Our HCV registry is hospital-based and may not reflect general population characteristics. Our survey can only show a part of DAA refusal and can help us adjust the policy and targeted groups of treatment refusal.

## 5. Conclusions

A significant proportion of patients with HCV infection refuse DAA treatment despite the Taiwan NHI coverage. The reasons for treatment refusal are multifactorial. The present findings can support policies for increasing access to HCV treatment.

## Figures and Tables

**Table 1:** Characteristics of patients with hepatitis C infection who have and have not received treatment.

	DAAs (N = 3566)	No DAAs (N = 418)	*p*-Value
Age (years)	65.7 ± 12.7	65.4 ± 16.3	0.712
Sex			0.481
Female	1984 (55.6)	225 (53.8)	
Male	1582 (44.4)	193 (46.2)	
Hemoglobin (g/dL)	13.2 ± 2	12.7 ± 2.3	<0.001
Platelet (10^3^/μL)	187.7 ± 70.3	209.4 ± 86.4	<0.001
WBC (10^3^/μL)	6.7 ± 2.6	7.2 ± 3.1	0.001
Prothrombin time (s)	11.9 ± 2.1	11.9 ± 2.4	0.981
AST (U/L)	35.5 ± 189.7	44.3 ± 79.2	0.357
ALT (U/L)	28.2 ± 88.6	40.5 ± 94.4	0.012
Creatinine (mg/dL)	1.2 ± 1.6	1.2 ± 1.4	0.974
FIB-4 score	3.4 ± 19.2	3 ± 3.7	0.727
HBV co-infection			<0.001
No	2367 (66.4)	342 (81.8)	
Yes	1199 (33.6)	76 (18.2)	
Cirrhosis	604 (16.9)	34 (8.1)	<0.001
Transfer time to hospital			0.044
<30 min	2599 (73.0)	327 (78.2)	
30–60 min	851 (23.9)	77 (18.4)	
>60 min	112 (3.1)	14 (3.3)	
Average (min)	24.8 ± 18.3	23.4 ± 20.1	0.138
Primary care physician visits *			<0.001
No	289 (8.1)	13 (3.1)	
Yes	3277 (91.9)	405 (96.9)	
Number	18.9 ± 30.7	13.9 ± 26.4	0.001
Gastroenterology visits *			<0.001
No	755 (21.7)	323 (77.3)	
Yes	2791 (78.3)	95 (22.7)	
Number	5.1 ± 4.3	0.8 ± 3.1	<0.001
Long-term drug use *	7.8 ± 9.6	7.3 ± 11.7	0.388

* With clinic visit record in 2019. ALT, alanine aminotransferase; AST, aspartate aminotransferase; FIB-4, Fibrosis-4 Index; HBV, hepatitis B virus; WBC, mean white blood cell count.

**Table 2:** Crude and adjusted odds ratios of HCV patients who received DAA treatment or not.

	Crude OR (95% CI)	*p*-Value	Adjusted OR (95% CI)	*p*-Value
Age (years)	1.00 (0.99–1.01)	0.655	1.002 (0.992–1.013)	0.699
Sex				
Female	ref.		ref.	
Male	0.93 (0.76–1.14)	0.482	0.64 (0.48–0.85)	0.002
Hemoglobin (g/dL)	1.12 (1.07–1.17)	<0.001	1.20 (1.12–1.29)	<0.001
Platelet (10^3^/μL)	0.996 (0.995–0.997)	<0.001	0.998 (0.996–1.000)	0.014
WBC (10^3^/μL)	0.93 (0.90–0.97)	<0.001	0.98 (0.93–1.02)	0.311
Prothrombin time (s)	1.00 (0.95–1.06)	0.980	0.996 (0.940–1.056)	0.897
AST (U/L)	1.000 (0.999–1.000)	0.398	0.999 (0.998–1.001)	0.410
ALT (U/L)	0.999 (0.998–1.000)	0.057	0.999 (0.997–1.001)	0.323
Creatinine (mg/dL)	1.00 (0.94–1.07)	0.974	1.04 (0.95–1.13)	0.435
HBV co-infection				
No	ref.		ref.	
Yes	2.28 (1.76–2.95)	<0.001	1.85 (1.35–2.54)	<0.001
Cirrhosis				
No	ref.		ref.	
Yes	2.30 (1.60–3.31)	<0.001	1.06 (0.67–1.66)	0.814
Average time to hospital				
<30 min	ref.		ref.	
30–60 min	1.39 (1.07–1.80)	0.013	1.40 (0.86–2.30)	0.179
>60 min	1.01 (0.57–1.78)	0.982	0.78 (0.21–2.93)	0.717
Any primary care physician visits *				
No	ref		ref.	
Yes	0.36 (0.21–0.64)	<0.001	0.07 (0.04–0.14)	<0.001
Gastroenterology visits *				
No	ref.		ref.	
Yes	12.24 (9.61–15.60)	<0.001	4.56 (2.82–7.37)	<0.001

Ref.: not-received HCV treatment; * with clinic visit record in 2019. ALT, alanine aminotransferase; AST, aspartate aminotransferase; FIB-4, Fibrosis-4 Index; HBV, hepatitis B virus; WBC, mean white blood cell count.

## Data Availability

The data presented in this study are available on request from the corresponding author.
